# Synthesized Nanorods Hydroxyapatite by Microwave-Assisted Technology for In Vitro Osteoporotic Bone Regeneration through Wnt/β-Catenin Pathway

**DOI:** 10.3390/ma14195823

**Published:** 2021-10-05

**Authors:** Nadia Z. Shaban, Marwa Y. Kenawy, Nahla A. Taha, Mona M. Abd El-Latif, Doaa A. Ghareeb

**Affiliations:** 1 Biochemistry Department, Faculty of Science, Alexandria University, Alexandria 21511, Egypt; nshaban2001@yahoo.co.uk (N.Z.S.); d.ghareeb@yahoo.com (D.A.G.); 2 Fabrication Technology Researches Department, Advanced Technology and New Materials Research Institute (ATNMRI), City of Scientific Research and Technological Applications (SRTA-City), New Borg El-Arab 21934, Egypt; amona1911@yahoo.com; 3 Modeling and Simulation Research Department, Advanced Technology and New Materials Research Institute (ATNMRI), City of Scientific Research and Technological Applications (SRTA-City), New Borg El-Arab 21934, Egypt; nahlataha_1982@yahoo.com; 4 Bio-Screening and Preclinical Trial Lab, Biochemistry Department, Faculty of Science, Alexandria University, Alexandria 21511, Egypt; 5 Center of Excellence for Drug Preclinical studies (CE-DPS), Pharmaceutical and Fermentation Industries Development Center (PFIDC), City of Scientific Research and Technological Applications (SRTA-City), New Borg El Arab 21934, Egypt

**Keywords:** biomaterials, bioceramics, sintering, glucocorticoid, osteoporosis, osteoblast, osteoclast, wnt pathway

## Abstract

This research presents an optimal and inexpensive, without any additives, method for the synthesis and sintering of hydroxyapatite (HA) by microwave-assisted technology (MAT) furnace. The target sintering temperature of the furnace (1100 ℃) was held for one and two hours for conventional sintering. With regard to the microwave hybrid sintering, it was held at 100%MW for 20 and 30 min. FTIR, XRD, TGA, SEM/EDS, and TEM were assessed to determine HA phase composition, and structural as well as thermal decomposition behavior. The in vitro effects of sintered HA discs on cultured aged mice-isolated osteoblast cells and hydrocortisone-induced osteoclast cells were assessed by measuring ALP, osteocalcin, TRAP, calcium, and Alizarin red S staining. Moreover, their effects on cell differentiation (CD90 and CD 105 and PARR- ɣ) and death markers (GSK3b, MAPK, and β-catenin) were evaluated. The results demonstrate the production of ≈35 nm crystal-sized pure hydroxyapatite nanorod-like particles with a high degree of crystallinity and no impurities as required for biomedical application. HA increased osteogenesis (ALP, osteocalcin, and calcium) markers and decreased cell resorption markers. In addition, HA nanorods reversed the effect of cortisone on cell differentiation and death markers. In conclusion, microwave hybrid sintered HA is a potential nanomaterial for osteoporotic bone regeneration as HA reversed the cortisone adverse effect on osteoblast cell death through canonical and non-canonical pathways.

## 1. Introduction

Bone is a mineralized connective tissue, containing about 69 wt % mineral phase (hydroxyapatite, needle-shaped), a 22 wt % organic phase (∼90 wt % type I collagen, ∼5 wt % noncollagenous proteins (NCPs), ∼2 wt % lipids), and 9 wt % water [1,2]. Bone displays four types of cells: osteoblasts, bone lining cells, osteocytes, and osteoclasts [3].

The most common metabolic bone disorder is osteoporosis, which is defined as bone quantity reduction. Worldwide, osteoporosis is a major fundamental cause of fractures in individuals over the age of 50 years and 200 million people are affected [4]. In the USA, the incidence of osteoporosis increased by 17.7% during the period 2004–2013 [5]. According to the statistical analysis of the Egyptian Osteoporosis Prevention Society, osteoporosis affects one in three women and one in five men. In Egypt, 53.9% of postmenopausal women have osteopenia and 28.4% have osteoporosis [6]. By 2050, the number of patients will increase annually by 21.3 million [4] as the number of elderly patients (over 50 years) will increase dramatically. For instance, the number in Australia will increase by 4% until it reaches 40 in the next years while in Arabic countries this percent will range between 30 and 40% [4].

Half of the survivors are incapable to walk, and 25% are kept in long-term care in a nursing home and this influences the economy as by 2050 about 80–90% of these patients will be untreated due to the several financial burdens [4,5]. Osteoporosis is characterized by low bone mass and bone microarchitecture deterioration due to the more rapid bone resorption process than bone formation, which usually leads to bone fragility and increased risks of fractures [6].

The most common available strategies used in preventing and treating osteoporosis are the use of agents that inhibit osteoclast activity hence bone resorption. The widely used antiresorptive agents are estrogen, selective estrogen receptor modulators (SERMs), bisphosphonates, and calcitonin. However, these agents cannot promote bone formation and are incapable of osteoporotic bone regeneration. Therefore, the inductive factors or osteoprogenitor cells during bone grafts are recently used to improve osteogenesis in osteoporosis [7,8].

Glucocorticoids have a crucial role in the normal regulation of bone remodeling, but prolonged glucocorticoid overexposure causes osteoporosis. In vivo, glucocorticoids promote bone resorption by stimulating osteoclastogenesis and consequently decreasing bone formation. Even though glucocorticoids promote in vitro osteoblastic cell differentiation, they have important inhibitory actions on bone formation [9,10].

Bioceramics are a viable material with a large number of clinical uses which go from head to toe and include bones, joints, and teeth repairs. These repairs become necessary when the existing part becomes diseased, damaged, or simply wears out [11]. One of the bioceramic materials is hydroxyapatite (HA), which is a member of the apatite group of ceramics. Hydroxyapatite bioceramic (Ca_10_(PO_4_)_6_(OH)_2_) with a hexagonal crystalline structure has been extensively studied due to its chemical similarity to the mineral component of bones.

The degree of crystallinity, crystallite size, processing condition (temperature, pressure, and partial water pressure), and porosity are considered as the factors affecting in vitro HA ionization and the dissolution rate [12]. HA has favorable biological properties that are proven in vitro and in vivo, which include biocompatibility, bioactivity, osteoconduction, osteointegration, and osteoinduction (in certain conditions) that resulted in rapid bone formation in a host body and strong biological fixation to bony tissues. Therefore, it represents a large number of regenerative graft materials are available in the market such as bone substitutes, metallic implants coat, tissue engineering scaffolds, and drug delivery carriers [11,13]. HA promoted the adhesion and osteogenic differentiation of cultured-osteoblast cells on their surface through Wnt/β- catenin signaling [14]. Wnt/β-catenin signaling plays a critical role in the achievement of peak bone mass, affecting the commitment of mesenchymal progenitors to the osteoblast lineage and the anabolic capacity of osteoblasts depositing bone matrix [15].

Different forms of HA can be used for biomedical applications as porous and dense blocks, granules, paste, cement, nanorods, belts, and coatings [16]. The nano-rods HA are better than other forms because they not only reduce bone loss but also increase cortical bone thickness.

Various methods are used to synthesize nanostructure HA powder including spontaneous combustion method [17], precipitation method [18], electrochemical deposition [19], sol–gel processes [20], hydrothermal synthesis [21], and microwave irradiation synthesis [2,22].

Microwave irradiation is a mild, convenient, rapid, and efficient method to synthesize HA, which results in the production of high-purity powder with small-narrow distributed particle sizes [23,24].

Sintering is a process of consolidating a compacted powder by heat treatment at elevated temperatures, usually at T > 0.5 Tm [K], where (Tm) is the melting temperature of that specific material. The sintering process aims to produce advanced sintered ceramic with microstructural control which is a high relative density and homogeneous microstructure of small grains [25]. Nano-powders are typically pressed and sintered to produce dense nanostructured ceramic materials. The high sintering temperatures and long sintering times required for the consolidation of ceramic powders frequently result in extreme grain coarsening and decomposition of the ceramic, which is typical of conventional sintering methods and leads to the deterioration of the mechanical properties [26]. To overcome the problem of grain growth, unconventional sintering and densification techniques have been proposed.

The hybrid heating technique combines direct microwave heating with radiant heating. The hybrid heating system will heat the sample more readily at low temperatures providing a more uniform heating gradient by flattening out the inverted temperature profile inside the ceramic body [27].

Hybrid microwave heating technique achieved by microwave assist technology (MAT) ovens combines the microwave and conventional radiation to heat materials rapidly and evenly. The hybrid heating system will heat the sample more readily at lower temperatures providing a more uniform heating gradient by flattening out the inverted temperature profile within the ceramic body, thus inhibiting grain growth, reducing the time and energy required during this formation process and improving the mechanical properties [27,28].

The present study was attempted to synthesize hydroxyapatite nanorod powder by microwave irradiation method. Then, nanorod hydroxyapatite is sintered in two methods: conventional heating and hybrid microwave heating at a temperature of 1100 ℃ and at different times. The second aim was to investigate the effect of nanorod hydroxyapatite before and after conventional and microwave hybrid sintering process on osteoporosis induced by cortisone in osteoblastic cells isolated from rat bone marrow through the Wnt/β-catenin signaling pathway.

## 2. Materials and Methods

### 2.1. Materials

Calcium nitrate tetrahydrate (Ca (NO_3_)_2_.4H_2_O) was purchased from Winter Laboratory (WinLab), UK (assay 99%—molar mass 236.15 g·mol^−1^). Ortho-phosphoric acid (o-H_3_PO_4_) was obtained from Sigma-Aldrich, St. Louis, MO, USA, (assay ≥85 wt. % in H_2_O—molar mass 98 g·mol^−1^). Sodium hydroxide (NaOH) was purchased from Fluka, Switzerland (assay 97%—molar mass 40 g·mol^−1^), hydrocortisone (C_21_H_30_O_5_) was obtained from Sigma-Aldrich, USA (assay ≥98%). Collagenase NB 4 Standard Grade was purchased from SERVA, Heidelberg, Germany (activity: ≥ 0.10 U/mg); ascorbic acid (Vitamin C) (USB Corporation Cleveland, St. Louis, MO, USA); minimum essential medium Eagle—alpha modification (α-MEM) Lonza, Belgium; Dulbecco’s modified Eagle’s medium, with 4.5 g/L glucose and L–glutamine (DMEM) (Lonza, Bornem, Belgium); L-glutamine; penicillin 10,000 IU/mL and streptomycin 10,000 μg/mL Lonza, Belgium fetal bovine serum (FBS) (heat-inactivated, EuroClone SpA, Pero, Italy); trypsin-EDTA solution containing 0.25% trypsin (Lonza, Belgium); dimethyl sulfoxide (DMSO) for freezing cells; 3-(4, 5-dimethylthiazol-2-yl)-2, 5-diphenyl tetrazolium bromide (MTT) obtained from BIO BASIC, Canada.

### 2.2. Animal and Ethical Approval

Male neonatal Wister albino rats (10 ± 5 g, aged 0 to 5 days) were obtained from experimental animal house, Institute of Graduate Studies and Research—IGSR, Alexandria University. All animal procedures were performed in the animal house of Pharmaceutical and Fermentation Industry Development Center (PFIDC), City of Scientific Research & Technological Applications (SRTA-city), New Borg El Arab, Alexandria.

This study design was approved by PFIDC institutional animal care and use committees (IACUSs) where the approval number was IACUC#34-3C-0719.

### 2.3. Synthesis of Nanorods HA Samples

Nano-rods HA powder sample was prepared by microwave-irradiation method via a home model microwave [22,29]. Total of 100 mL of H_3_PO_4_ (0.1 M) solution was heated until 60 °C then a 100 mL of Ca(NO_3_)_2_·4H_2_O (0.167 M) solution was added until the temperature reached 60 °C again. The pH of the mixture solution was adjusted to 11 with about 10 mL of NaOH (5M) solution to obtain a homogeneous milky solution using magnetic stirring in all steps. After that, a 200 mL of homogeneous milky solution added in a 1000 mL beaker for every single preparation was heated in a 26 L home model microwave chamber 2.45 GHz, 900 W (CA MW2626, CAIRA, Egypt) for 30 min until dehydration. Then, the precipitate was heated in a drying oven (Nabertherm controller B170, Germany) at 100 °C for 30 min until complete drying. The overall chemical reaction occurred as follows:

10 (Ca(NO_3_)_2_·4H_2_O) + 6 H_3_PO_4_ + 20NaOH → Ca_10_ (PO_4_)_6_ (OH)_2_ (↓) + 20 NaNO_3_ + 58 H_2_O

The resulting white precipitate was washed with distilled water several times and then dried at 100 °C for 8 h. The obtained HA powder was named HA green (HAg). The HA white powder was dry pressed with mold pressure 80 MPa to form HA discs. HA discs were checked using a digital micrometer for a uniform thickness of 0.8 mm and a diameter of 1.0 mm.

### 2.4. Sintering of HA Discs

Hydroxyapatite discs were sintered with two different techniques: conventional sintering and microwave hybrid sintering using a microwave assist technology (C-MAT) furnace (MRF 16/22 Carbolite, Derbyshire, UK). The prepared HA discs (two samples per firing cycle/method) were put in an Alundum ignition ceramic boat inside the C-MAT furnace cavity. The furnace temperature was raised by a heating rate of 5 °C/min until the target sintering temperatures reached 1100 °C. For Conventional sintering: the temperatures were held for 1–2 h at 1100 °C and for Hybrid Microwave sintering: the microwave was switched on using 100% MW power, then held for 20–30 min. Subsequently, the furnace was allowed to cool using furnace cooling fans to room temperature by the cooling rate. Each optimized sintered HA disc was checked using a digital micrometer to confirm the uniform shrinkage of samples [30,31].

### 2.5. Characterization of HA Samples

The green HA and HA sintered disc samples were coated with gold and investigated microscopically using scanning electron microscopy (SEM, JEOL JSM 6360LA, Tokyo, Japan) coupled with energy dispersive X-ray spectroscopy (EDX, Gatan, Pleasanton, CA, USA), for each HA sample (*n* = 3). The microscopic size of the HA green sample was characterized by transmission electron microscopy (TEM, JEOL 2100 PLUS, Tokyo, Japan). Thermal stability of the HA green sample was studied using a thermogravimetric analyzer (TGA-50, Shimadzu, Japan). The temperature program was raised from room temperature to 1200 °C with a heating rate of 10 °C/min under nitrogen flow rate of 20 mL/min [32]. Fourier-transform infrared spectroscopy (FTIR-8400 S Shimadzu, Japan) was used to determine the functional groups of the HA samples. The recorded region is from 400–4000 cm^−1^ using the spectroscopic grade potassium bromide (KBr) pellet technique. The crystal phases of the HA green and sintered samples will be obtained using an X-ray diffractometer (XRD—6100 Shimadzu, Japan), operating with CuKα radiation (λ = 0.154060 nm) generated at 30 kV and 30 mA, scans were performed at 2° min^−1^ for 2θ values between 20 and 60 degrees. XRD data were used to calculate crystallite size and crystallinity [33,34]. The mean crystallite size (D) of the particles in nm was calculated from the line broadening measurement of XRD reflection from Scherrer’s equation [22]:

$$D\left(\mathrm{nm}\right)=\frac{0.89\;\lambda }{\beta \;\cos\;\theta },$$

where: λ = 0.15405 is the wavelength for (CuKα), *β* the full width at half-maximum for the diffraction peak under consideration (rad), and θ is Bragg’s diffraction angle (°). The diffraction peak at 2 θ = 26.04° was chosen for the calculation of the crystallite size since it was much sharper and isolated from the others. This peak was assigned to (0 0 2) Miller’s plane family and showed the crystal growth along the c-axis of the hydroxyapatite crystalline structure.

The degree of crystalline phase (*X*_c_) of HA powders was evaluated by the following equation [35]:

$$X_{c}=\frac{1-V_{112/300}}{I_{300}},$$

where: *I*_300_ is the intensity of (3 0 0) diffraction peak and *V*_112/300_ is the intensity of the hollow between the characteristic peaks of the planes (1 1 2) and (3 0 0) of HA.

The mechanical testing of HA samples: For the determination of the surface roughness values, a portable surface roughness tester (Mitutoyo Surftest, SJ-201, Kawasaki, Japan) was used. It is a hand-held electronic instrument that measures the peak-to-valley height of the surface profile of cleaned and polished surfaces. Three discs were measured for each sintered samples to obtain the mean roughness value [36]. After completing the surface roughness testing, the same samples were used for determination of the hardness values. Hardness evaluation was conducted using Digital Microhardness Tester (Zwick/Roell, IDENTEC, ZHVμ-S, West Midlands, England), by applying a load of 50 g and 500 g (for HAg and HA different sintered discs, respectively) for 10 s. Each sample was positioned in a manner that the device’s indenter tip was perpendicular to the sample surface to be tested. The average of the five indentations was then calculated for each sample. The mean of the three examined samples for each sintered discs was taken as the hardness of the material [37].

### 2.6. Isolation of Osteoblast Cells and Induction of Osteoclast Cell Formation

Rat calvarial osteoblast (RCO) cultures were used as a model to assess osteoblast behavior and differentiation. Cells were isolated from 0 to 5 days old neonatal male Wister albino rats. Parietal and occipital bones were dissected and washed with phosphate-buffered saline (PBS). The harvest bones were minced and subsequently digested by incubating in 700 units/mL of type 1 collagenase at 37 °C. The supernatant of the first digestion was discarded and the calvarial fragments were treated five times with collagenase (20 min at 37 °C) and the subsequent supernatants were collected, combined, and sedimented. The resulting cell pellet was re-suspended and cultured in cultured media (CM) α-MEM supplemented with 10% (*v*/*v*) fetal bovine serum (FBS), and antibiotics (200 U/mL penicillin and 200 µg/mL streptomycin). RCO cultures were maintained in a humidified 5% CO_2_ atmosphere at 37 °C [38].

### 2.7. Induction of Osteoclast Cell Formation (Induction of Osteoporosis)

Induction of osteoporosis using glucocorticoids was carried out according to the protocols of Ishida and Heersche (1998) and Weinstein (2012) with some modifications [10,39]. Osteoblastic cells were plated into six-well sterile culture plates at 10^5^ cells/well and incubated in CM until a confluent layer was achieved. To determine the dose of cortisol used in this experiment, osteoblastic cells were plated in sterile six-well culture plates at 10^5^ cells/well incubated in CM until a confluent layer was achieved and a series concentration of hydrocortisone (0, 0.125, 0.25, 0.5, 1, 2 mg/mL) was added. The plate was incubated for 21 days, then the concentration of Tartrate-resistant acid phosphatase (TRAcP) was measured in each well, and it was found that TRAcP was increased in constant value in a concentration of 0.5–2 mg/mL, therefore we chose 0.5 mg/mL cortisone to induced osteoclast formation. To a confluent layer of osteoblast cells, 0.5 mg/mL cortisone was added and every 3–4 days the media was changed. After 21 days, the existence of osteoclast cells was assessed by 10% alizarin red S stain.

### 2.8. Culture of Osteoblast (OB) and Osteoclast (OC) on HA Discs

The osteoblast or osteoclast cells were cultured on six-well sterile culture plates at a concentration of 1 × 10^5^ cells/well. Cells were allowed to grow in complete media (10% FBS, 2 mM L-glutamine in α-MEM) for 2 days to reach 70% confluence. Thereafter, the spent media were removed and replaced with fresh media (2 mL of α-MEM) containing 6.8 mg different HA discs (HAg, HA1h, HA2h, HA20m, and HA30m), each HA disc cleaned in an ultrasonication bath and autoclaved before using). Cells were incubated at 37 °C, 5% CO_2_ for 7 days. The adherent cells were enzymatically detached with trypsin/EDTA solution (1X) and then the suspended cells were collected by centrifugation at 400× *g* for 5 min. The cell pellets were washed three times with phosphate buffer saline (PBS). Then, cells were resuspended in PBS (pH 7.4) containing protease inhibitor and subjected to ultrasonication three times. The supernatant was collected for quantification of biochemical parameters [40,41].

### 2.9. Cell Viability and Proliferation Test

Different HA samples (6.8 mg/mL) were plated in a 96-well cell culture plate with 200 μL of the cultured medium that contained 100,000 osteoblasts or osteoclast/well in DMEM medium followed by plate incubation for 7 days in a CO_2_ incubator (37 °C, 5% CO_2_, and 90% relative humidity). At the end of the treatment period, MTT was first prepared as a stock solution of 5 mg/mL in phosphate buffer saline PBS (pH 7.2) and filtered. Then, 20 μL of MTT solution was added to each well and the plate was incubated for 4 h in a CO_2_ incubator. After incubation, the plate was centrifuged at 1650 rpm for 10 min and the medium was discarded. The formazan crystals (MTT byproduct) were re-suspended in 100 μL DMSO and reading was measured at a wavelength of 570 nm [42].

The % viability was calculated as follows: (AT/AC) × 100.

AT **=** mean absorbance of cells treated with different HA samples.

AC **=** mean absorbance of control untreated cells with culture medium only.

### 2.10. Mineralization Detection

Alizarin red S staining was used to evaluate the successful isolation of osteoblast cells and in vitro bone-formation which produced calcium-rich deposits. The cells were carefully washed with PBS, pH 7.4. After PBS aspiration, cells were fixed in neutral buffered formalin (10%) and incubated for 30 min. After formalin solution removal, enough alizarin red S staining solution (1%, pH 4.1–4.3) was added to cover the cell monolayer and the plate was incubated at room temperature in the dark for 45 min. Carefully, alizarin red S staining solution was aspirated and the cell monolayer was washed four times with distilled water. Cells were examined under the inverted microscope with 400× magnification. Calcium deposits were stained bright orange-red [43].

### 2.11. Bone Remodeling Biomarkers

Proliferation markers such as osteocalcin, alkaline phosphatase (ALP), and extracellular calcium concentration were assayed using commercial kits supplied from Nordic Bioscience Diagnostics (Herlev, Denmark), Biosystems (Barcelona, Spain), and Sigma- Aldrich (St. Louis, MO, USA), respectively, while Tartrate-resistant acid phosphatase (TRAcP) as a marker of bone resorption was assayed according to Takara Bio Inc (Kusatsu, Japan).

### 2.12. Molecular Investigations

The levels of CD105, β-catenin, GSK3β, and GSK3β pS9 were measured by manual quantitative ELISA technique using rabbit polyclonal CD105 (# PA5-80582, Invitrogen, Waltham, MA, USA), rabbit monoclonal β-catenin, and GSK3β (#8480 and #9315, respectively, Cell Signaling Technology, Danvers, MA, USA) as well as rabbit polyclonal GSK3β pS9 (#abx328236, abbexa, Shanghai, China). The antigen was diluted to a final concentration of 100 µg protein in a coating buffer (bicarbonate/carbonate, 0.2 M, pH 9.6) in wells of polyvinyl chloride (PVC) microtiter plate. Samples were loaded in duplicate and incubated overnight at 4 °C. The remaining protein binding sites in the coated wells were blocked using blocking solution (bovine serum albumin (BSA), 5%). To reduce the non-specific binding, primary and secondary antibodies were diluted in the blocking solution. After incubation with primary and secondary antibodies (Goat anti-rabbit IgG, ALP, #A8025, Sigma-Aldrich, USA), substrate solution (ρ-nitrophenyl phosphate, disodium salt, PNPP) was added. The reaction was stopped using sodium hydroxide (3 M) and the color developed was read at 450 nm on a plate reader (Sanofi Diagnostics Pasteur, Lyon, France). Standard curves for each protein were constructed [44]. Determination of CD90, PARR-ɣ, and p38-MAPK proteins levels were determined using an ELISA kit according to manufactures’ instructions. The concentration of GSK3β and GSK3β pS9 were expressed in the µg/mg protein while p38 MAPK, CD90, and CD105 concentrations were expressed in ng/mg protein and PARR-ɣ, and β-catenin concentrations were expressed in the pg/mg protein.

### 2.13. Statistical Analysis

Data were analyzed by one-way analysis of variance (ANOVA) using Primer of Biostatistics (Version 5) software program using SPSS Statistics (StatSoft, Tulsa, OK, USA). The significance of means ± SD was detected groups by the Student-Newman-Keuls comparison test at *p* < 0.05.

## 3. Results and Discussion

### 3.1. Chemical Analysis of Hydroxyapatite Samples

#### 3.1.1. X-ray Diffraction (XRD) Analysis

**Figure 1:**
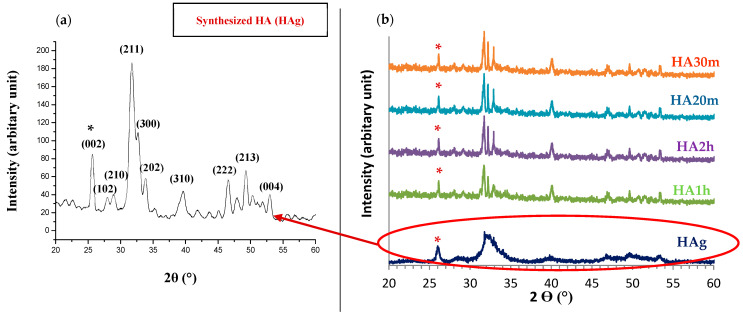
X-ray diffraction pattern of the hydroxyapatite samples: (**a**) XRD spectra of hydroxyapatite nanoparticles (HA green) sample (**b**) HA green, HA microwave hybrid sintered at 1100 °C for 20 min (HA20m) and 30 min (HA30m) and HA conventional sintered at 1100 °C for 1 h (HA1h) and 2 h (HA2h), respectively. (*) the (002) reflection peak.

The XRD pattern of the HA powder prepared by the microwave irradiation method (HAg) shows that all the peaks were indexed according to the hexagonal phase of hydroxyapatite and the standard XRD card (ICDD No. 01-074-0565) as displayed in Figure 1a. While Figure 1b shows the effect of different sintering temperatures/time on the prepared hydroxyapatite, the diffraction peaks of sintered samples were narrow and well separated and had the highest intensity when compared to the broad diffraction peaks of the green HA sample. The position and FWHM (full width at half-maximum) of the (002) reflection peak were used for the calculation of crystal size and crystallinity index. Table 1 shows that we succeed in producing ≈35 nm crystal-sized pure hydroxyapatite nanorods-like particles with a high degree of crystallinity and no impurities as required for biomedical application. The sintering time in both sintering techniques (conventional and hybrid microwave sintering) had an increasing effect on hydroxyapatite average crystallite size and HA crystallinity index. In contrast, HA samples sintered by hybrid microwave heating showed an increase in the intensity of peaks than that of the samples sintered by conventional heating. Furthermore, the intensity of this peak increased when the time of sintering was increased. Based on the XRD measurements, no impurity peaks such as calcium oxide (CaO) or β-TCP were detected in the diffraction pattern before and after the heat treatment process. This indicates that all HA samples were phase-purified and that both used sintering methods did not cause hydroxyapatite decomposition. Therefore, it is acceptable to use our HA samples as biomaterials in biomedical applications [33,45].

#### 3.1.2. Fourier Transform Infrared Spectra of HA Powder

**Figure 2:**
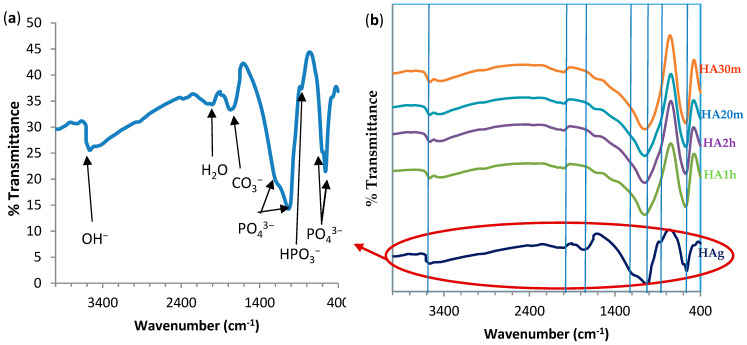
FTIR spectra of hydroxyapatite: (**a**) Hydroxyapatite green sample, (**b**) HA green, HA conventional sintered at 1100 °C for 1 h (HA1h) and 2 h (HA2h), respectively, and HA microwave hybrid sintered at 1100 °C for 20 min (HA20m) and 30 min (HA30m) respectively.

Figure 2 shows the FTIR spectrum of precipitated HA powder before and after heat treatments. Figure 2a shows the typical absorption bands for PO_4_^3−^ at 559.3–560 cm^−1^, 878 cm^−1^, and 1040 and 1196 cm^−1^ [46]. There was a broadband of H_2_O at 1768 cm^−1^. Likewise, there was a band at 636 cm^−1^ and an extended vibration of the ions around 3578 cm^−1^ due to hydrogen-bonded (OH-) and a typical C=O band at 1653 cm^−1^. These were the characteristic absorption bands of pure hydroxyapatite powders [47,48].

Effects of sintering temperatures on the FTIR spectra of prepared hydroxyapatite samples: The typical property of PO_4_^3−^ bands of apatite structure at (559 and 1040 cm^−1^) was identified in all HA sintered samples. In addition, the peak of the PO_4_^3−^ band in 876 cm^−1^ gradually decreased in intensity and sharpened the band with increasing heat treatment time. While the PO_4_^3−^ band at 636 cm^−1^ appeared in the HAg sample only and disappeared in all sintered samples. In contrast, the intensity of the ion stretching band around 3578 cm^−1^ for hydrogen-bonded (OH-) gradually decreased with increasing sintering time as this band is very “sensitive” to temperature changes. All peaks were more involved in the conventional sintered and hybrid microwave samples than that in the green hydroxyapatite sample (HAg). The peak of the C=O band at 1638 cm^−1^ decreased significantly with increasing sintering temperatures. These results are in agreement with the previous work of A. Rapacz-Kmita, et al. 2005, which proves that dehydroxylation of hydroxyapatite that occurs at conventional sintering was more than that of hybrid microwave sintering [47].

#### 3.1.3. Thermal Stability Analysis of HA Powder

**Figure 3:**
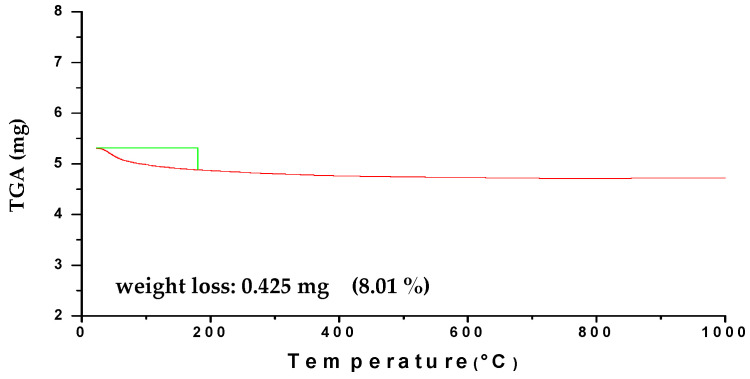
Thermal analysis pattern of green HA powder.

The TGA thermogram of green HA powder obtained by microwave irradiation method in the temperature range of 20–1200 °C as demonstrated in Figure 3. The weight loss diagram plotted a characteristic region at 22.95–182.38 °C, and the weight loss was about 0.425 mg (8.01%) which was attributed to moisture vaporization (liberation of chemically bonded water). There was no significant weight loss in the range of 500–1200 °C where a stable curve was noticed within this temperature range, which confirms that the prepared HAg samples were thermally stable at high temperatures [32]. Thus, the prepared HA is a good material for sintering at high-temperature 1100 °C and suitable for pharmaceutical and biomedical processing at 37 °C.

#### 3.1.4. Microstructure of HA Powder Using Transmission Electron Microscope

**Figure 4:**
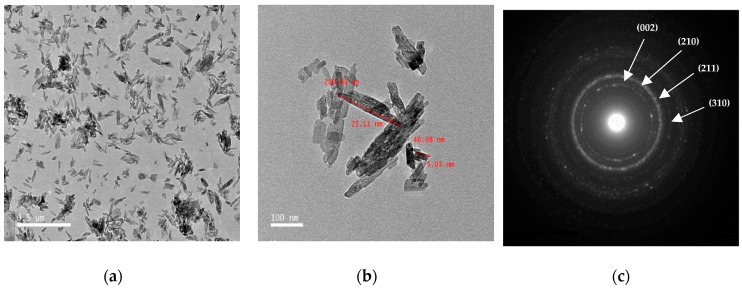
Transmission electron microscope (**a**,**b**) TEM micrograph of nanorods HA particles at different fields, showing HA nano rod-like particles, and (**c**) SAED pattern.

TEM micrograph and the selected area electron diffraction (SAED) pattern of HA green particles are shown in Figure 4a−c respectively. It is observed that green HA was a rod-shaped morphology and smooth agglomerates of the crystal are also displayed in Figure 4a. The crystallite size of HAg is estimated from TEM images by image analysis freely available ImageJ software (ImageJA, GitHub, San Francisco, CA, USA) and the estimated average crystallite dimension was 13.67 ± 5 nm width and 102.05 ± 10 nm height (mean ± SD) as demonstrated in Figure 4b. The SAED pattern in Figure 4c confirms that the crystalline structure of HA was hexagonal and this is compatible with our XRD results [32,37].

#### 3.1.5. Scanning Electronic Micrograph of HA Powder

**Figure 5:**
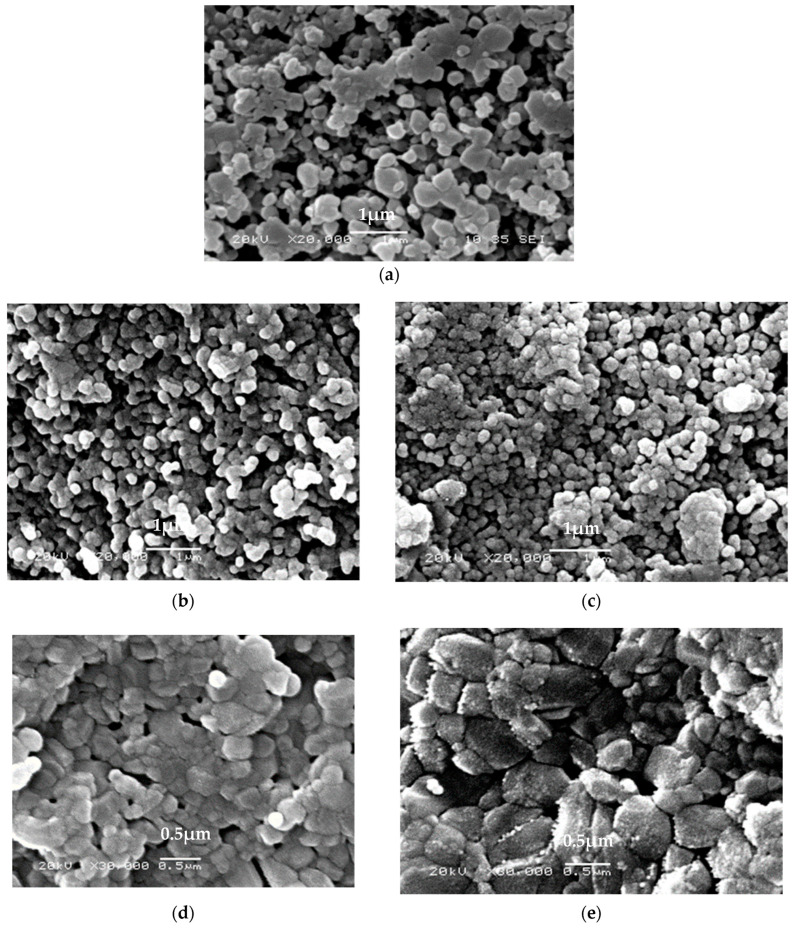
Morphology of hydroxyapatite (**a**) HAg: HA green without heat treatment, (**b**,**c**) HA microwave hybrid sintered at 1100 °C for 20 min (HA20m) and 30 min (HA30m) respectively. And (**d**,**e**) HA conventional sintered for 1 h (HA1h) and 2 h (HA2h) respectively.

The morphological study of HA particles was studied using scanning electron microscope (SEM) as shown in Figure 5. The HA powder appeared agglomerated and consisted of a large number of fine HA particles with a relatively large pore size as shown in Figure 5a. The effects of sintering temperatures on the morphological features of prepared HA samples were characterized with sintering time variations of each sintering technique at 20,000× and 30,000× magnification. However, there was a significant increase in particle size of sintered conventional HA samples as compared to the sintered HAg and microwave samples. While there was uniform grain growth along with decreased porosity and clear grain boundaries in the microwave sintered samples. In general, this increase in particle size may be due to the augmentation of the coalescence of particles in conventional sintering. Whereas in the hybrid microwave sintering, the grain size was decreased and almost all sample porosity removed, which indicates that almost full-density HA was produced from both sintering techniques. These results were also found by S. Aarthy et al. [49].

#### 3.1.6. The Elemental Compositions of HA Powder

Elemental analysis technology was performed to determine the elemental composition of HA samples. In this study, we evaluated the Ca/P concentration ratio of different HA samples using SEM/EDX. EDX is a sensitive qualitative and semi quantitative analytical technique on the other hand EDX may be a destructive analysis in many cases. In general, the analytical resolution depends on the incident beam energy, the critical excitation energy for the X-rays of interest, the atomic weight, the atomic number, and the density of the sample which are mentioned in details in the ISO standard “ISO 22309:2011 Microbeam analysis—Quantitative analysis using energy-dispersive spectrometry (EDS) for elements with an atomic number of 11 (Na) or above”.

The standard EDX spectra recorded on the examined HA is shown in Table 2. The presented data show that the Ca/P atomic ratio of the prepared HA was 1.658. The effect of sintering temperature and time on nano-hydroxyapatite samples was recorded using EDX spectra. In general, by increasing the sintered time, the Ca/P atomic ratio was increased until reaching the stoichiometric value of (≈1.67) in microwave hybrid sintered samples (HA20m and HA30m). Comparatively, the atomic Ca/P ratio (1.70) of HA2h was the highest. Based on these results and SEM images, it is hypothesized that this increase in the atomic ratio in conventional and microwave hybrid sintering may be due to the coalescence of these particles occurring in three successive steps, approximation of particles, the formation of necks at particles contacts, and the coalescence of these particles. This has been previously demonstrated by S. Raynaud, et al. and J.K. Abifarin et al. [50,51].

#### 3.1.7. The Mechanical Properties of HA Discs

The influence of sintering temperature and time on the surface roughness and Vickers hardness of HA discs before and after sintered by conventional and microwave techniques are shown in Table 3. The values of surface roughness of HA discs ranged between 0.87 and 1.89 µm. The HAg showed the highest surface roughness (1.89 µm). The surface roughness was decreased with the increase in the sintering time in both sintering techniques at 1100 ℃. In the literature, Deligianni et. al. 2000 reported a roughness value of HA ranging between 0.73 and 4.68 µm and they showed that the roughness obtained on the HA surface had macroscopically parallel grooves and ridges. Thus, elongated cells (like osteoblast cells) orientated along grooves even on the smoother surfaces (low surface roughness) [36].

The mean Vickers microhardness of the green HA discs was 16 HV (0.16 GPa). These results are in agreement with that reported by Yudyanto et al. 2019 who stated that the hardness of the prepared HA was 10 HV and the differences in the microhardness may be due to the different preparation methods [52]. Considering different HA sintering techniques, the linear increase of the Vickers microhardness with increasing sintering time was noted. The maximum of 263 HV (2.58 GPa) was achieved at the conventional sintered HA2h sample. This result was in agreement with the findings of Curran et al. 2011 who reported that the hardness of their conventional and microwave sintered HA increased with increasing grain size [53,54].

### 3.2. Effect of HA on Osteoporotic Bone Regeneration

**Figure 6:**
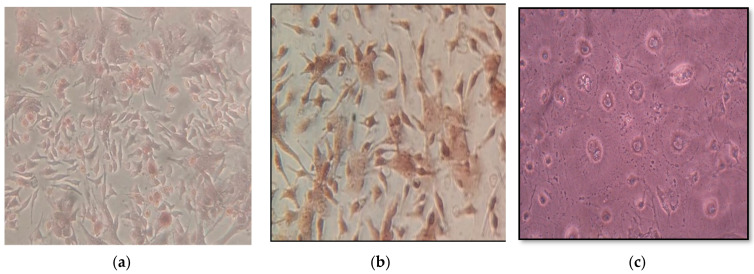
Inverted microscopy of alizarin red S-stained osteoblastic cells: Cells at day 21 of culture, (**a**,**b**) the differentiated osteoblasts cells show vast extracellular calcium deposits, stained in bright reddish and the oblong trapezoid-shaped morphology of the osteoblast cells with 200× and 400× magnifications respectively; (**c**) osteoclast cells (without extracellular calcium deposits) larger, multinucleated cells seen in a cluster rather than singly with 400× magnification.

Figure 6 shows that osteoblast cells (with extracellular calcium deposits) were bright reddish while the osteoclast cells had no color (without extracellular calcium deposits) after 21 days of culture. Furthermore, it is noted that the osteoblast cells had oblong trapezoid-shaped morphology displaying positive staining for calcium deposition. While the osteoclast cells were larger, multinucleated, and appeared as a cluster. These results indicate the success of the isolation of osteoblast cells from the long bone of rats by type 1 collagenase digestion as well as the addition of hydrocortisone-induced osteoblast cells death and osteoclast cells formation [55,56].

#### 3.2.1. Effect of HA Treatment on Biological Characteristics of Osteoblast and Osteoclast Cells

**Figure 7:**
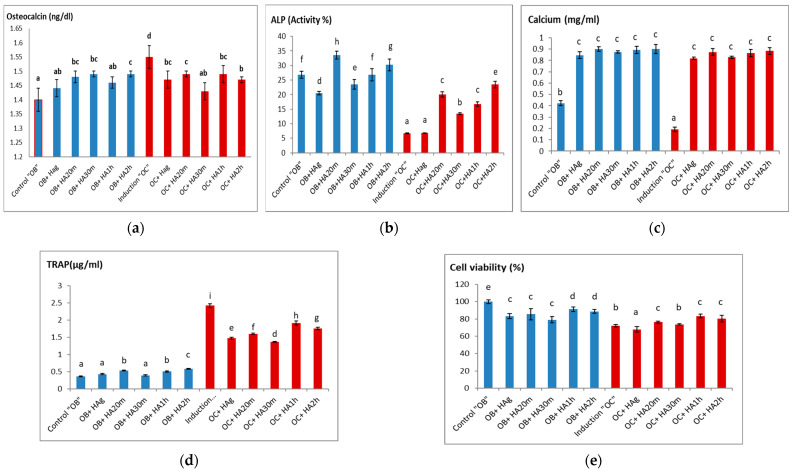
The effect of different HA discs on (The response of osteoblast and osteoclast cells to different HA discs treatment): (**a**) osteocalcin concentration, (**b**) alkaline phosphatase (ALP) concentration, (**c**) intracellular calcium concentration, (**d**) tartrate-resistant acid phosphatase (TRAcP) concentration, (**e**) cell viability. In each bar, means with different letters are significantly different from each other where (a) the lowest mean, means the same letters are significantly similar at *p* < 0.05.

Figure 7 shows the effect of hydroxyapatite disc treatment on bone formation markers (osteocalcin and ALP), extracellular calcium concentration, bone resorption marker (TRAP), and cell viability, respectively. As demonstrated in Figure 7a, concerning the control (OB cells) group that showed the lowest osteocalcin level, treatment with cortisol for 21 days (OC cells) increased the osteocalcin to its maximum obtained value, at *p* > 0.05. Although all OB treatments increased the osteocalcin levels, they decreased OC osteocalcin levels, at *p* < 0.05. 

Treatment of osteoblast with HAg and HA30m decreased the ALP activity while the treatment with HA1h did not affect the enzyme activity and finally, the treatment of OB with HA20m and HA2h increased the enzyme activity when compared to OB level, at *p* < 0.05. Where OB treated with HA20m showed the highest ALP activity, at *p* < 0.05. For osteoclast cells that showed the lowest ALP activity, all treatments with different HA discs increased ALP activity to lower levels than that of OB except the treatment with HA g that showed OC activity, at *p* < 0.05 as shown in Figure 7b.

OC cells showed the lowest calcium concentration, *p* < 0.05. Treatment of OB or OC cells with different HA discs had a significantly increased calcium level higher than that of the OB cells, and these positive increments in all treated cells were significantly similar, at *p* < 0.05 (Figure 7c). Treatment of OB cells with HA20m, HA1h, and HA2h significantly increased TRAPc activities than that of control (OB) cells, while the other treatment showed the same control OB level, at *p* < 0.05. In the case of OC cells which showed the highest TRAP level, addition of HA different discs significantly decreased TRAP levels but failed to normalize them at *p* < 0.05. Among the OC-treated groups, the lowest level appeared in the case of HA30m disc at *p* < 0.05 (Figure 7d). Finally, Figure 7e showed that the OB and OC cells were seeded on the HA scaffolds surface and cultured for seven days, then a viability test was performed. Treatment of OB cells with different HA discs significantly decreased cell viability than that of control (OB) cells at *p* < 0.05. In the case of OC cells, cell treatment with HAg decreased cell viability while treatment with HA1h and HA2h increased it, and finally the treatment with HA20m or HA30m did not affect the proportion of cell viability at *p* < 0.05.

As previously mentioned in the literature, osteoblastic activity is associated with serum osteocalcin, which is one of the proteins found in a relatively high concentration in bone. Osteoblast produces osteocalcin protein and a very small amount is released into the circulation [57]. Glucocorticoids administration cause osteoblast cells death that is associated with the liberation of the cellular content into serum and consequently an increase of osteocalcin level [58]. 

The HA ceramics addition increases the adhesion of osteoblast to their surface and stimulates the osteogenesis as well as prevents osteoblastic cell death by glucocorticoid leading to a decrease in osteocalcin level [59]. The ALP enzyme which is a membrane-bound enzyme plays an important role in bone mineralization through the hydrolysis of phosphate esters as well as pyrophosphates which are accounted as inhibitors of bone mineralization. During active bone formation, the level of ALP which is a byproduct of osteoblast activity is increased [60]. When HA discs were added to cells, calcium concentration is increased due to the dynamic dissolution of calcium ions from hydroxyapatite. These results are consistent with the theory reported in Tang Z. et al. (2018). The extracellular calcium ions Ca^2+^ could act as a carrier of biological signals for bone marrow progenitor cells or premature osteoblast cells at the site of bone resorption after which it stimulates these progenitor cells’ maturation into osteoblast cells to produce new bone [60].

TRAcP is an enzyme specifically expressed in high amounts by osteoclasts cells and has been evaluated as a bone resorption marker. Therefore, the elevated level of TRAcP in OC indicates the formation of osteoclast cells and the occurrence of bone resorption mechanism [61]. This also proves that glucocorticoid addition induces osteoblast death and osteoclast formation. Moreover, Wang et al. (2004) and Costa et al. (2013) proved that the HA ceramics sintered at higher temperature significantly enhance osteoblast function by inducing the bone formation markers while suppressing the osteoclast activity [40,62]. Costa et al. (2013) reported that hydroxyapatite ceramics negatively induce the TRAcP level [40]. Furthermore, it is mentioned that the HA ceramics sintered at higher temperatures and for a long time affected cell proliferation [62]. Furthermore, changes in the surface topography of hydroxyapatite after sintering influence cell differentiation and proliferation [40].

#### 3.2.2. Effect of Hydroxyapatite Treatment in Bone-MSC and Osteoblast Death Signals

Table 4 shows that all treatments of OB cells did not affect bone mesenchymal cell (MSCs—are precursors of the osteoblasts) signals (CD90 and CD 105 as well as PARR- ɣ) and osteoblast death cell signals (GSK3βpS9, GSK3β, MAPK, and β-catenin) compared to control OB cells at *p* < 0.05. Furthermore, the ratio of OB cells treated with different HA discs showed the same GSK3βpS9/GSK3β level except for the treatment with HA20m that showed the lowest ratio level at *p* < 0.05. Addition of cortisone increased mesenchymal stem cells (MSCs) markers (CD90 and CD 105) as well as adipocytes marker (PARR- ɣ) and cellular death markers (GSK3βpS9, GSK3β, MAPK, β-catenin, and the ratio GSK3βpS9/GSK3β) to their maximum levels at *p* < 0.05. The treatment of OC cells with different HA discs decreased all tested markers and the GSK3βpS9/GSK3β ratio when compared to OC levels. However, all these parameters were higher than that of OB cells at *p* < 0.05. MSCs are progenitor cells that subsequently differentiate into mature adipocytes or mature osteoblasts. Ishida and Heersche 1998 along with our study results showed that the effectiveness of glucocorticoid depends on GCs concentrations i.e., low concentrations of GCs stimulate osteoblast differentiation and increase bone formation. While elevated concentrations of GCs stimulate osteoclast formation and osteoblast apoptosis [39]. Therefore, the elevated concentration of cortisone differentiates the MSCs into adipocytes and participates in osteoclast formation. The treatment with all different HA discs carried on osteoclastic cells decreased CD90, CD 105, and PARR- ɣ, indicating the differentiation of MSC into osteoblast or the prevention of osteoclast formation.

Moreover, the addition of cortisone to osteoblast cells for 21 days increased GSK3β, GSK3βpS9, and MAPK. Several studies show that osteoporosis formation increased GSK3β induction and phosphorylation (GSK3βpS9) which in turn phosphorylates and accumulates β-catenin in cell cytoplasm finally inhibiting the Wnt pathway [14,15]. Moreover, the induction of MAPK stimulates the formation of PARP- ɣ that activates MSC differentiation into adipocytes. Moreover, MAPK phosphorylates GSK3β which in turn phosphorylates and accumulates β-catenin leading to osteoclast formation and osteoblasts cell death [59,63]. Altogether, cortisone not only affects the Wnt/catenin pathway but also induces inflammation microenvironment which promoted β-catenin accumulation. Moreover, β-catenin accumulation occurs when adipocytes formation takes place to suppress the noncanonical Wnt/Ca(^2+^) pathway which consequently arrests osteoblast formation and reduces the osteogenic differentiation. Treatment with all different HA discs conducted on OC improved the tested parameters, nonetheless, did not normalize them as mentioned before by several authors who stated that the treatment with different calcium/phosphorus forms induced bone formation through Wnt/catenin pathway [14,15]. Finally, the obtained results proved that the treatment with the microwave hybrid sintered HA (HA20m) was the best at *p* < 0.05.

## 4. Conclusions

Microwave hybrid sintering speeds up heating processes, saves energy, and improves chemical and biochemical properties of nanorod hydroxyapatite compared to conventional sintering. Microwave hybrid sintering cause a uniform growth of HA grains with highest crystallinity (88.72%) at HA30m. Moreover, the Ca/P atomic ratio was increased until reaching the stoichiometric value of (≈1.67) in microwave hybrid sintered samples (HA20m and HA30m). Hydrocortisone can suppress osteoblast function and thus reduce bone remodeling and impair bone tissue repair. These occur by increasing apoptosis in osteoblasts through the production of reactive oxygen species (ROS), inhibition of bone formation transcription factors, and accumulation of β-catenin leading to suppression of the Wnt/β-catenin pathway. Nanorod hydroxyapatite enhanced osteoblast functions proliferation, alkaline phosphates, calcium synthesis, bone formation transcription factors production that correlate with decreased β-catenin accumulation leading to activation of the Wnt/β-catenin pathway. The resorptive activity of osteoclasts cultured with nanorod HA was weaker than that of control osteoclastic cells. In addition, cell adhesion and proliferation were enhanced by the presence of HA. Therefore, microwave hybrid sintered nanorod hydroxyapatite may serve as a valuable scaffold for osteoporotic bone regeneration.

## Figures and Tables

**Table 1:** Average crystallite size and crystallinity of hydroxyapatite.

Sample	Sintering Time	Average Crystallite Size (nm)	Average Crystallinity (%)
HAg	-	34.9	75.09
HA1h	1 h	45.7	87.31
HA2h	2 h	45.6	86.69
HA20m	20 min	37.9	85.87
HA30m	30 min	39.1	88.72

**Table 2:** Elemental composition of the sintered hydroxyapatite samples.

Sample	Sintering Time	Elements/At (%)				Ca/P Ratio
		O	Ca	P	C	
**HAg**	**-**	56.92 ± 0.3	24.79 ± 0.1	14.95 ± 0.5	3.34 ± 0.2	1.658
HA1h	1 h	50.80 ± 0.1	23.35 ± 0.1	14.02 ± 0.5	11.83 ± 0.1	1.665
HA2h	2 h	50.40 ± 0.5	23.82 ± 0.3	14.00 ± 0.6	11.78 ± 0.2	1.701
HA20m	20 min	50.90 ± 0.7	24.43 ± 0.1	14.60 ± 0.3	10.07 ± 0.1	1.673
HA30m	30 min	47.69 ± 0.2	23.34 ± 0.2	13.91 ± 0.7	15.06 ± 0.3	1.678

Results are the means ± SD in each sample.

**Table 3:** Statistical analysis of surface roughness (μm) and Vickers microhardness (HV and GPa) of HA samples.

Sample	Sintering Time	Surface Roughness R_a_ (μm)	Vickers Microhardness	
			(HV)	(GPa)
HAg	-	1.89 ± 0.1	16 ± 0.2	0.16 ± 0.1
HA1h	1 h	0.98 ± 0.1	229 ± 2.9	2.25 ± 0.2
HA2h	2 h	0.87 ± 0.1	263 ± 3.8	2.58 ± 0.3
HA20m	20 min	1.69 ± 0.2	104 ± 4.3	1.02 ± 0.1
HA30m	30 min	1.67 ± 0.1	135 ± 5.6	1.32 ± 0.1

Results are the means ± SD in each sample.

**Table 4:** Effect of different HA discs on bone-MSC and osteoblast death markers of cortisone-induced osteoclast formation.

Groups		CD90 (ng/mg)	CD105 (ng/mg)	PARR- ɣ (pg/mg)	P38-MAPK (ng/mg)	GSK3βpS9 (µg/mg)	GSK3β (µg/mg)	GSK3βpS9 /GSK3β	β.catenin (pg/mg)
Control OB		3.21 ± 0.01 ^A^	1.48 ± 0.07 ^A^	16.44 ± 1.3 ^A^	0.35 ± 0.01 ^A^	3.3 ± 0.02 ^A^	5.6 ± 0.13 ^A^	0.59 ± 0.06 ^B^	1.35 ± 0.005 ^A^
OB	HAg	3.21 ± 0.02 ^A^	1.53 ± 0.02 ^A^	16.93 ± 2.3 ^A^	0.33 ± 0.02 ^A^	3.2 ± 0.05 ^A^	5.3 ± 0.12 ^A^	0.60 ± 0.07 ^B^	1.36 ± 0.030 ^A^
OB	HA20m	3.23 ± 0.02 ^A^	1.46 ± 0.09 ^A^	17.62 ± 0.9 ^A^	0.32 ± 0.03 ^A^	2.8 ± 0.09 ^A^	5.6 ± 0.21 ^A^	0.500 ± 0.07 ^A^	1.37 ± 0.007 ^A^
OB	HA30m	3.19 ± 0.02 ^A^	1.58 ± 0.05 ^A^	16.23 ± 1.6 ^A^	0.34 ± 0.02 ^A^	3.1 ± 0.01 ^A^	5.1 ± 0.17 ^A^	0.61 ± 0.04 ^B^	1.32 ± 0.003 ^A^
OB	HA1h	3.23 ± 0.01 ^A^	1.51 ± 0.20 ^A^	15.31 ± 1.8 ^A^	0.36 ± 0.01 ^A^	3.3 ± 0.01 ^A^	5.5 ± 0.15 ^A^	0.60 ± 0.08 ^B^	1.38 ± 0.001 ^A^
OB	HA2h	3.21 ± 0.03 ^A^	1.45 ± 0.08 ^A^	16.13 ± 2.8 ^A^	0.39 ± 0.04 ^A^	3.4 ± 0.11 ^A^	5.9 ± 0.23 ^A^	0.58 ± 0.04 ^B^	1.36 ± 0.003 ^A^
Induction OC		7.32 ± 0.08 ^G^	6.85 ± 0.32 ^F^	54.24 ± 1.6 ^D^	1.73 ± 0.12 ^F^	15.7 ± 1.2 ^E^	11.9 ± 1.80 ^E^	1.32 ± 0.09 ^G^	3.97 ± 0.023 ^F^
OC	HAg	6.20 ± 0.31 ^F^	5.11 ± 0.24 ^D^	42.31 ± 2.7 ^C^	1.63 ± 0.23 ^E^	11.3 ± 2.3 ^D^	14.3 ± 1.10 ^D^	0.790 ± 0.04 ^F^	3.72 ± 0.120 ^E^
OC	HA20m	3.94 ± 0.07 ^B^	2.73 ± 0.21 ^B^	20.36 ± 0.8 ^B^	0.65 ± 0.03 ^B^	6.1 ± 0.21 ^B^	8.92 ± 0.30 ^B^	0.68 ± 0.01 ^C^	1.98 ± 0.008 ^B^
OC	HA30m	4.08 ± 0.04 ^C^	3.01 ± 0.19 ^B^	18.73 ± 3.1 ^AB^	0.77 ± 0.02 ^C^	7.9 ± 0.32 ^C^	10.9 ± 0.50 ^C^	0.72 ± 0.02 ^D^	2.1 ± 0.003 ^C^
OC	HA1h	4.98 ± 0.05 ^D^	4.17 ± 0.34 ^C^	18.64 ± 0.9 ^AB^	0.93 ± 0.07 ^D^	9.3 ± 0.78 ^D^	11.9 ± 0.70 ^C^	0.78 ± 0.03 ^E^	2.87 ± 0.009 ^D^
OC	HA2h	5.92 ± 0.10 ^E^	4.93 ± 0.27 ^D^	40.83 ± 3.7 ^C^	1.61 ± 0.21 ^E^	10.8 ± 1.8 ^D^	13.6 ± 0.90 ^D^	0.79 ± 0.01 ^F^	3.63 ± 0.015 ^E^

The results are the means ± SD in each group. In each column, means with different letters (^A^, ^B^, ^C^, ^D^, ^E^ and, ^F^) are significantly different from each other where (^A^) the lowest mean, means with the same letters are significantly similar at *p* < 0.05. OB is osteoblast cells, and OC is osteoclast cells.

## Data Availability

The data presented in this study are contained within the article.
